# Sp1 Plays a Key Role in Vasculogenic Mimicry of Human Prostate Cancer Cells

**DOI:** 10.3390/ijms23031321

**Published:** 2022-01-25

**Authors:** Deok-Soo Han, Eun-Ok Lee

**Affiliations:** 1 Department of Science in Korean Medicine, Graduate School, College of Korean Medicine, Kyung Hee University, 26, Kyungheedae-ro, Dongdaemun-gu, Seoul 02447, Korea; ejr0957@khu.ac.kr; 2 Department of Cancer Preventive Material Development, Graduate School, College of Korean Medicine, Kyung Hee University, 26, Kyungheedae-ro, Dongdaemun-gu, Seoul 02447, Korea

**Keywords:** vasculogenic mimicry, Sp1, human prostate cancer cells, twist, VE-cadherin, AKT

## Abstract

Sp1 transcription factor regulates genes involved in various phenomena of tumor progression. Vasculogenic mimicry (VM) is the alternative neovascularization by aggressive tumor cells. However, there is no evidence of the relationship between Sp1 and VM. This study investigated whether and how Sp1 plays a crucial role in the process of VM in human prostate cancer (PCa) cell lines, PC-3 and DU145. A cell viability assay and three-dimensional culture VM tube formation assay were performed. Protein and mRNA expression levels were detected by Western blot and reverse transcriptase-polymerase chain reaction, respectively. The nuclear twist expression was observed by immunofluorescence assay. A co-immunoprecipitation assay was performed. Mithramycin A (MiA) and Sp1 siRNA significantly decreased serum-induced VM, whereas Sp1 overexpression caused a significant induction of VM. Serum-upregulated vascular endothelial cadherin (VE-cadherin) protein and mRNA expression levels were decreased after MiA treatment or Sp1 silencing. The protein expression and the nuclear localization of twist were increased by serum, which was effectively inhibited after MiA treatment or Sp1 silencing. The interaction between Sp1 and twist was reduced by MiA. On the contrary, Sp1 overexpression enhanced VE-cadherin and twist expressions. Serum phosphorylated AKT and raised matrix metalloproteinase-2 (MMP-2) and laminin subunit 5 gamma-2 (LAMC2) expressions. MiA or Sp1 silencing impaired these effects. However, Sp1 overexpression upregulated phosphor-AKT, MMP-2 and LAMC2 expressions. Serum-upregulated Sp1 was significantly reduced by an AKT inhibitor, wortmannin. These results demonstrate that Sp1 mediates VM formation through interacting with the twist/VE-cadherin/AKT pathway in human PCa cells.

## 1. Introduction

Prostate cancer (PCa) is a common cancer among men around the world [1]. PCa spread to nearby organs, tissues and other parts of the body including lymph nodes and bones [2]. After spreading, cancer cells attach to the other tissues and grow to form new tumors that can cause damage where they land [3]. Reportedly, a quarter of men with PCa in the world have a metastatic disease and the 5-year survival rate of patients with metastasis to distant sites is 29% [4]. PCa cells are known to have largely aggressive properties [5]. Since these tumors need a blood supply to grow and spread through blood circulation [6], it is important to shut off the blood supply to prevent tumor growth and metastasis in PCa.

Vasculogenic mimicry (VM), discovered in 1999 [7], is the alternative neovascularization by aggressive tumor cells without the presence of endothelial cells (ECs) and it functions as blood vessels by ECs [8,9,10]. Blood supply is the indispensable process for cancer cells to grow and metastasize through providing oxygen and nutrients [11]. However, the therapeutic efficacy of drugs targeting only blood vessels by ECs is limited due to an adequate blood supply through a new pattern such as VM [12,13,14]. VM has appeared in various types of cancer including PCa and is related to poor prognosis of cancer patients by meta-analysis [15,16]. Overall survival (OS) and disease-free survival (DFS) were significantly lower in VM-positive PCa patients [17]. Since VM has the essential effects on tumor progression, VM is a new therapeutic strategy to improve the therapeutic efficacy of cancer patients including PCa. 

Sp1 transcription factor is overexpressed in many types of cancer cells including PCa and controls several genes that are involved in many cellular processes, including cell differentiation, cell growth, apoptosis, angiogenesis, and response to DNA damage [18,19,20,21]. Additionally, it contributes to progression and metastasis of PCa [21]. Therefore, Sp1 is an attractive target of cancer treatment in PCa patients. Although there are many studies on the functions of Sp1, there is no evidence of the relationship between Sp1 and VM formation. Among human PCa cell lines, PC-3 and DU145 cells have a powerful property of VM formation compared with LNCaP cells [22]. Thus, this study investigated whether and how Sp1 affects VM formation in human PCa PC-3 and DU145 cells.

## 2. Results

### 2.1. Sp1 Mediates VM Formation in PCa Cells

**Figure 1:**
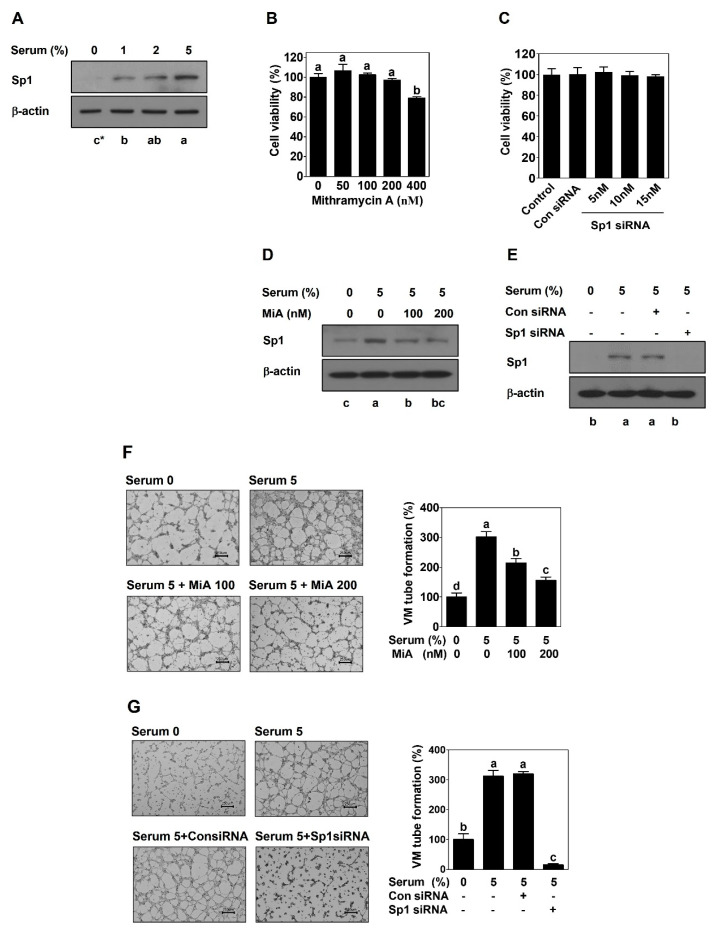
Sp1 is upregulated by serum and mediates VM formation in PC-3 cells. Western blot was performed in serum-treated cells (**A**), in MiA-treated with cells with serum (**D**), and in siRNA-transfected cells with serum (**E**) for 24 h. Cell viability was measured by MTT assay in MiA-treated with cells with serum (**B**), and in siRNA-transfected cells with serum (**C**) for 24 h. VM tube formation assay was carried out in MiA-treated cells with serum (**F**) and in siRNA-transfected cells with serum (**G**). After 16 h incubation, images were obtained under an inverted light microscope at 40× magnification. Scale bar = 250 μm. The number of formed VM structures was counted. Data are shown as mean ± SD and were statistically calculated by one-way ANOVA followed by Tukey’s studentized range test. * Means with different letters are significantly different between groups.

PC-3 cells were treated with an increasing concentration of serum for 24 h and then the expression level of Sp1 was checked by Western blot. Sp1 was dramatically upregulated by serum in a dose-dependent manner (Figure 1A). Since serum promotes VM formation of PC-3 cells [23], to determine the role of Sp1 and VM formation, loss-of-function approach was introduced. PC-3 cells were treated with a selective Sp1 inhibitor, mithramycin A (MiA), or were transfected with the siRNA-targeting Sp1 gene. First, the cell viability assay was performed to determine non-cytotoxic concentrations of MiA and Sp1 siRNA. There was no cytotoxic effect of MiA or siRNA up to 200 nM or 15 nM, respectively (Figure 1B,C). This study used 100 and 200 nM of MiA or 15 nM of siRNA for subsequent experiments. Serum-upregulated Sp1 expression was effectively inhibited by MiA or Sp1 silencing (Figure 1D,E). To determine whether Sp1 is associated with VM formation, 3D culture VM formation assay was performed in PC-3 cells after MiA treatment or transfection with Sp1 siRNA. Serum stimulation led to the induction of tubular channels by PC-3 cells, which was effectively reduced by MiA in a dose-dependent manner (Figure 1F). Similarly, Sp1 silencing had an obvious inhibitory effect on serum-induced the formation of tubular channels (Figure 1G).

**Figure 2:**
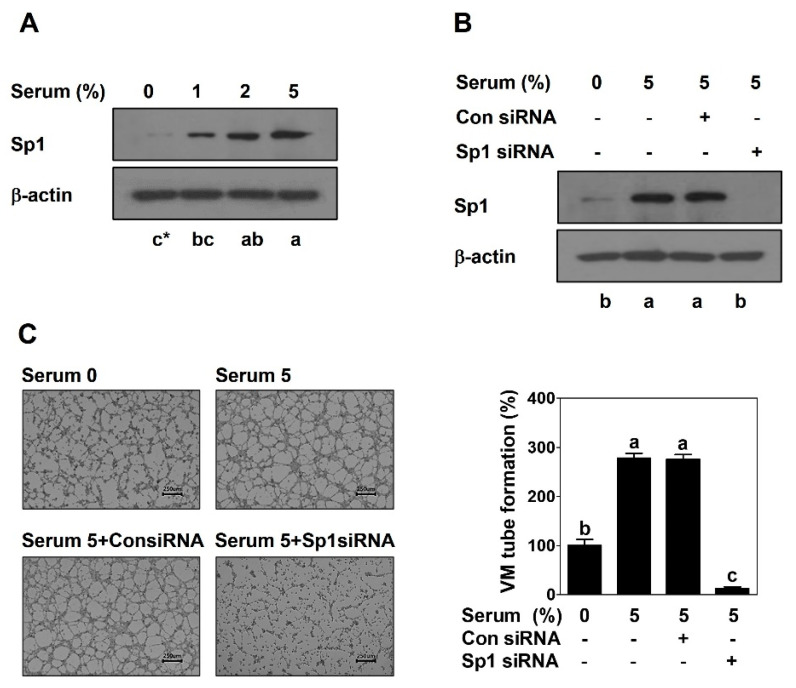
Sp1 is upregulated by serum and mediates VM formation in DU145 cells. Western blot was performed in serum-treated cells (**A**) and in siRNA-transfected cells with serum (**B**) for 24 h. (**C**) VM tube formation assay was carried out in siRNA-transfected cells with serum. After 16 h incubation, images were obtained under an inverted light microscope at 40× magnification. Scale bar = 250 μm. The number of formed VM structures was counted. Data are shown as mean ± SD and were statistically calculated by one-way ANOVA followed by Tukey’s studentized range test. * Means with different letters are significantly different between groups.

To verify a role of Sp1 in VM formation, a Western blot for Sp1 after serum treatment and transfection with Sp1 siRNA, and 3D culture VM formation assay after transfection with Sp1 siRNA were performed in another PCa DU145 cells. Consistent with the results from PC-3 cells, Sp1 was upregulated by serum in DU145 cells (Figure 2A). Additionally, serum-induced VM formation was significantly reduced after Sp1 silencing in DU145 cells (Figure 2B,C).

**Figure 3:**
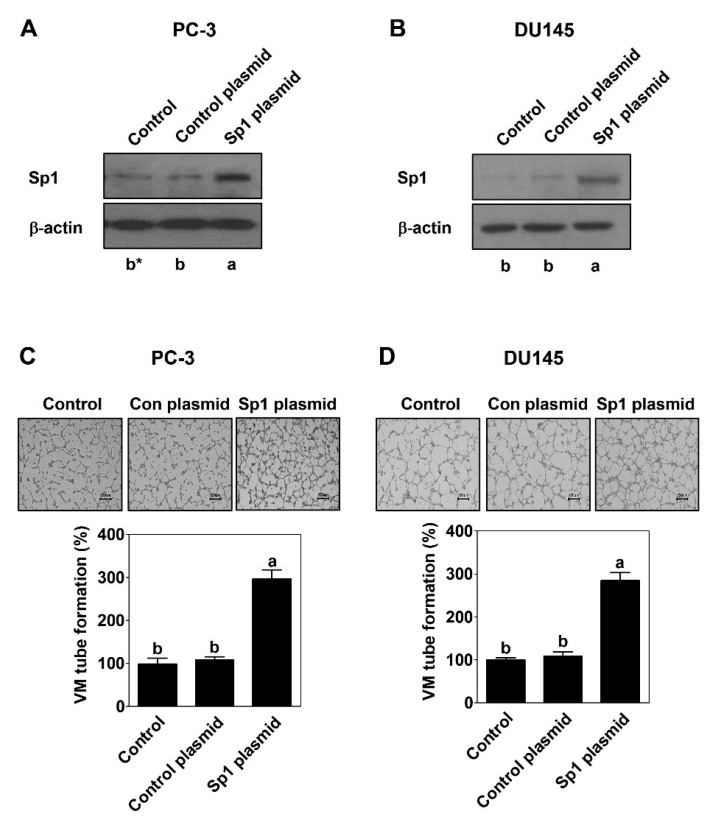
Sp1 mediates VM formation in PCa cells. Western blot was performed in PC-3 cells (**A**) and DU145 cells (**B**) after transfection with CRISPR activation plasmid. VM tube formation assay was carried out in PC-3 cells (**C**) and DU145 cells (**D**) after transfection with CRISPR activation plasmid. After 16 h incubation, images were obtained under an inverted light microscope at 40× magnification. Scale bar = 250 μm. The number of formed VM structures was counted. Data are shown as mean ± SD and were statistically calculated by one-way ANOVA followed by Tukey’s studentized range test. * Means with different letters are significantly different between groups.

To confirm a novel functional role of Sp1 in VM formation, a gain-of-function approach was introduced using Sp1 CRISPR activation plasmid in both PC-3 and DU145 PCa cells. Sp1 overexpression caused an effective increase in VM tubular formation compared with control plasmid without serum in both PC-3 (Figure 3A) and DU145 cells (Figure 3B) by a 3D culture VM formation assay.

Taken together, Sp1 silencing inhibited serum-stimulated VM formation, whereas Sp1 overexpression triggered VM formation in PCa cells, suggesting that Sp1 is required to induce VM formation in PCa cells.

### 2.2. Sp1 Upregulates VE-Cadherin Expression through the Nuclear Twist in PC-3 Cells

**Figure 4:**
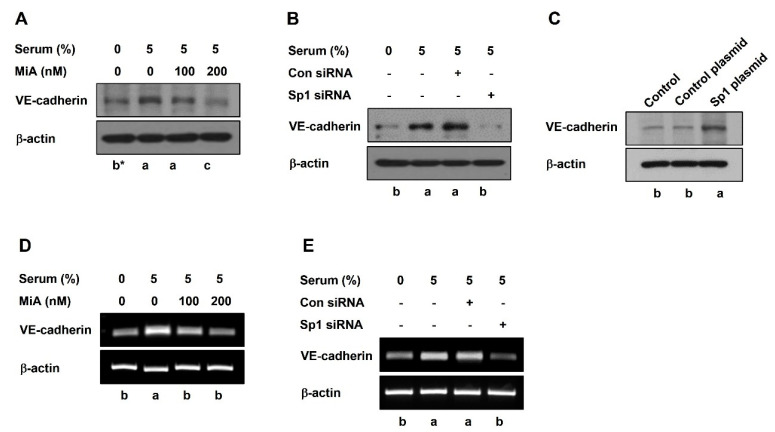
Sp1 regulates VE-cadherin expression at the transcription level in PC-3 cells. Western blot was performed in MiA-treated cells with serum (**A**) and in siRNA-transfected cells with serum (**B**) for 24 h, and in CRISPR activation plasmid-treated cells (**C**). mRNA level was analyzed by RT-PCR in MiA-treated cells with serum (**D**) and in siRNA-transfected cells with serum (**E**) for 24 h. Data were statistically calculated by one-way ANOVA followed by Tukey’s studentized range test. * Means with different letters are significantly different between groups.

To reveal whether Sp1 affects the expression of VE-cadherin to induce VM formation, a Western blot was conducted in PC-3 cells. Serum upregulated VE-cadherin protein expression, which was attenuated by MiA in a dose-dependent manner (Figure 4A). Additionally, VE-cadherin protein expression by serum was markedly inhibited in Sp1 siRNA-treated cells (Figure 4B). However, Sp1 overexpression slightly upregulated VE-cadherin protein expression without serum (Figure 4C). To assess whether the VE-cadherin protein level was affected by the transcriptional level, the mRNA expression level of VE-cadherin was detected by RT-PCR. Consistent with the protein expression of VE-cadherin, the serum-upregulated mRNA level of VE-cadherin was decreased after treatment with MiA (Figure 4D) or Sp1 siRNA (Figure 4E). These results indicated that Sp1 regulates VE-cadherin expression at the transcription level.

**Figure 5:**
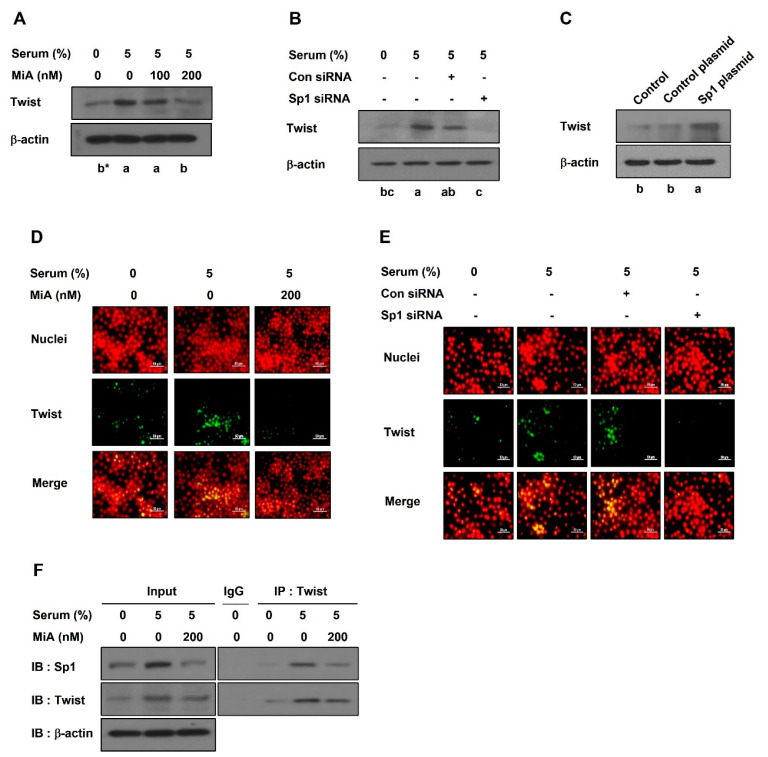
Sp1 enhances the nuclear localization of twist in PC-3 cells. Western blot was performed in MiA-treated cells with serum (**A**) and in siRNA-transfected cells with serum (**B**) for 24 h, and in CRISPR activation plasmid-treated cells (**C**). The nuclear twist expression was detected by immunofluorescence assay in MiA-treated cells with serum (**D**) and in siRNA-transfected cells with serum (**E**) for 24 h. After incubating with twist antibody (green) followed by FITC-conjugated secondary antibody, the nuclei were counterstained with propidium iodide (red). Images were obtained by a fluorescence microscope at 400× magnification. Scale bar = 40 μm. (**F**) Co-IP was performed in MiA-treated cells with serum. IgG: negative control. Data were statistically calculated by one-way ANOVA followed by Tukey’s studentized range test. * Means with different letters are significantly different between groups.

To identify the transcriptional regulation of VE-cadherin, a Western blot and immunofluorescence analysis were performed in PC-3 cells. Twist was elevated by serum, which was decreased by MiA treatment (Figure 5A) or Sp1 silencing (Figure 5B). However, the overexpression of Sp1 increased the expression level of twist without serum compared with control plasmid (Figure 5C). Immunofluorescence staining showed that enhanced twist expression in the nucleus by serum was attenuated after MiA treatment (Figure 5D) or Sp1 silencing (Figure 5E). As shown in Figure 5F, the interaction between Sp1 and twist was induced by serum, which was significantly reduced by MiA treatment. Taken together, these results demonstrated that the nuclear twist upregulates VE-cadherin expression, which the process of which is mediated by Sp1.

### 2.3. Sp1 Promotes the Activation of AKT Pathway in PC-3 Cells

**Figure 6:**
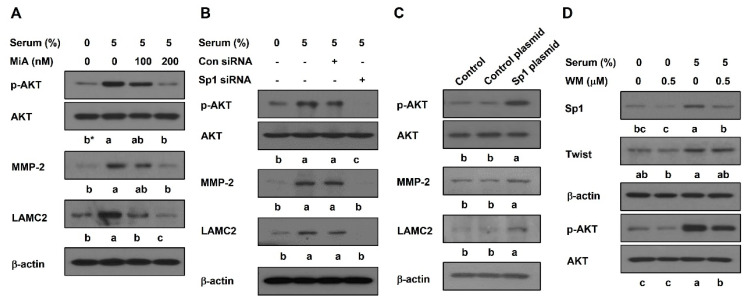
Sp1 promotes the activation of AKT pathway in PC-3 cells. Western blot was performed in MiA-treated cells with serum (**A**) and in siRNA-transfected cells with serum (**B**) for 24 h, in CRISPR activation plasmid-treated cells (**C**), and in serum-treated cells with or without wortmannin (WM) for 24 h (**D**). Data were statistically calculated by one-way ANOVA followed by Tukey’s studentized range test. * Means with different letters are significantly different between groups.

To investigate whether Sp1 is involved in the AKT pathway to induce VM formation, the Western blot analyzed PC-3 cells. The phosphorylation of AKT and the expression levels of MMP-2 and LAMC2 were augmented by serum. MiA treatment (Figure 6A) or Sp1 silencing (Figure 6B) decreased the effects of serum. In contrast, Sp1 overexpression elevated the phosphorylation of AKT and the expression levels of MMP-2 and LAMC2 without serum compared to the control plasmid (Figure 6C). Serum-upregulated Sp1, but not twist, was significantly reduced by the AKT inhibitor, wortmannin (Figure 6D). These results indicate that Sp1 contributes to the activation of VM-related AKT signaling. Additionally, Akt was controlled by Sp1 expression.

## 3. Discussion

VM is the formation of a vessel-like network lined by cancer cells. The function of VM is similar to that of blood vessels formed by ECs [8,9,10]. VM strongly participates in tumor invasion, metastasis, and growth through a blood supply and is closely related to poor prognosis in cancer patients [8,15,24]. VM-positive PCa patients showed high Gleason scores and distance metastasis as well as short OS and DFS [17]. The Sp1 transcription factor plays a crucial role in the progression and metastasis of PCa [21]. However, the involvement of Sp1 in VM formation has not been determined yet. Therefore, this study investigated a novel functional role of Sp1 in the process of VM in human PCa cells.

Since a previous study demonstrated that serum promotes VM formation in human PCa PC-3 cells [23], this study focused on Sp1 to explore an underlying molecular mechanism of VM. As expected, serum dramatically upregulated the expression of Sp1 at the protein level in both PCa PC-3 and DU145 cells (Figure 1A and Figure 2A). To elucidate a novel functional role of Sp1 in VM formation, MiA and Sp1 siRNA used for a loss-of-function approach and Sp1 CRISPR activation plasmid was used for a gain-of-function approach. The inhibition of Sp1 by MiA and Sp1 siRNA caused the perfect blockage in VM formation induced by serum (Figure 1 and Figure 2). On the contrary, despite the absence of serum, the overexpression of Sp1 by CRIPSR activation plasmid sufficiently formed VM (Figure 3). Therefore, these results clearly demonstrate that Sp1 may be an important factor in the process of VM formation in PCa cells.

Highly aggressive tumor cells overexpress VE-cadherin, but not non-aggressive tumor cells [25]. VE-cadherin, an endothelial-specific junction molecule, is a biomarker of VM and plays a crucial role in VM formation [26,27,28]. The endothelial-specific transcriptional active region of VE-cadherin contains the Sp1 binding site [29,30], highlighting the relationship between Sp1 and VE-cadherin. In this study, the serum-upregulated expressions of VE-cadherin at the protein and mRNA levels were decreased after treatment with MiA or Sp1 siRNA (Figure 4), highlighting the transcriptional regulation of VE-cadherin expression. However, the overexpression of Sp1 upregulated the protein expression of VE-cadherin (Figure 4C). Twist is a transcription factor that regulates the expression of VE-cadherin [31,32]. Twist has been reported to be associated with tumor metastasis and angiogenesis [33] and also regulates VM formation [32]. In this study, serum-treated PC-3 cells were found to increase the expression of twist in the nucleus, which was reduced by the inhibition of Sp1 by MiA or siRNA (Figure 5A,B). However, the overexpression of Sp1 elevated the protein expression of twist (Figure 5C). Sp1 interacted with twist, which was significantly reduced by MiA treatment (Figure 5F). Taken together, these results revealed that Sp1 regulates the expression of VE-cadherin by interacting with twist in the nucleus.

Multiple signaling pathways such as AKT, FAK, hypoxia, and nodal/notch contribute to VM formation [8,24]. Among them, as a downstream signaling of VE-cadherin, AKT is activated by VE-cadherin [8,34]. Then, activated AKT elevates the expressions of matrix metalloproteinases (MMPs) such as MMP-2 and -14, thereby leading to VM formation through the remodeling of the extracellular matrix including LAMC2 [8,24]. Additionally, AKT promotes cancer cell growth, proliferation, and malignant behavior [35]. A previous study demonstrated that the AKT/MMP-2/LAMC2 signal transduction pathway participates in VM formation in response to serum [23]. Sp1 knockdown suppressed tumor progression by inhibiting AKT and ERK signaling [36]. AKT-mediated VEGF mRNA expression required Sp1 [37]. These reports indicated that Sp1 may be involved in the AKT signaling pathway. In this study, the serum-induced phosphorylation of AKT in PC-3 cells was seen to decrease when Sp1 was suppressed by MiA or siRNA (Figure 6A,B). However, the overexpression of Sp1 enhanced the phosphorylation of AKT (Figure 6C). Meanwhile, AKT signaling also regulated the Sp1 expression. Both serum-upregulated MMP-2 and LAMC2 expressions were decreased when Sp1 was inhibited by MiA or siRNA (Figure 6A,B). On the contrary, the overexpression of Sp1 enhanced the expression levels of MMP-2 and LAMC2 (Figure 6C). These results verified that Sp1 is involved in the AKT pathway to induce VM in PC-3 cells.

**Figure 7:**
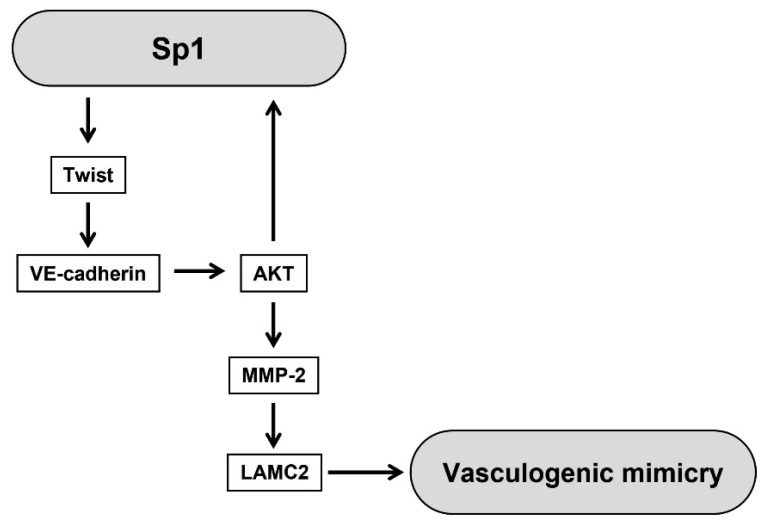
Proposed molecular mechanism for Sp1-induced vasculogenic mimicry in human PCa cells.

In conclusion, this study demonstrated a novel functional role of Sp1 in VM formation through loss- and gain-of-function approaches and these results are summarized in Figure 7. Sp1 regulated the expression of VE-cadherin through controlling the nuclear expression of transcription factor, twist. Sp1-induced the upregulation of twist/VE-cadherin in turn activated the AKT pathway including MMP-2 and LAMC2, thereby causing an induction of VM. Taken together, Sp1 plays a key role in VM formation through the twist/VE-cadherin/AKT pathway in human PCa cells. These results may provide a new therapeutic strategy for the treatment of PCa patients associated with VM through targeting Sp1.

## 4. Materials and Methods

### 4.1. Cell Culture

The human prostate cancer cell lines PC-3 and DU145 were purchased form Korean Cell Line Bank (KCLB, Seoul), and were grown in RPMI 1640 medium (Welgene Inc., Daegu, Korea) supplemented with 10% fetal bovine serum (FBS, Welgene Inc., Daegu, Korea) and 1% antibiotics (Welgene Inc., Daegu. Korea) in a humidified incubator at 37 °C with 5% CO_2_.

### 4.2. Sp1 Silencing by Small Interfering RNA (siRNA)

Cells (1 × 10^5^) were seeded on a 6-well plate and transfected with 15 nM of control or Sp1 siRNA (Santa Cruz Biotechnology, Inc., Danvers, MA, USA) for 48 h using INTERFERin transfection reagent (Polyplus-transfection Inc., New York, NY, USA) according to the manufacturer’s protocol.

### 4.3. Sp1 Overexpression by CRISPR Activation Plasmid

Cells (1.2 × 10^5^) were seeded on a 6-well plate and transfected with 1 μg of control or Sp1 CRISPR activation plasmid (Santa Cruz Biotechnology, Inc., Danvers, MA, USA) for 48 h using UltraCruz transfection reagent (Santa Cruz Biotechnology, Inc., Danvers, MA, USA) according to the manufacturer’s protocol.

### 4.4. Cell Viability Assay

Cells were seeded at 10,000 cells per well in a 96-well plate, and treated with various concentrations (50, 100, 200 and 400 nM) of mithramycin A (MiA, Enzo Life Sciences, Farmingdale, NY, USA) for 24 h in a serum-free culture medium. siRNA-transfected cells (2.5 × 10^3^) were seeded in a 96-well plate and transfected with control siRNA or various concentrations (5, 10 and 15 nM) of Sp1 siRNA for 48 h. The effects of MiA and siRNA on the cell viability of PC-3 cells were evaluated by 3-(4,5-dimethylthiazol-2-yl)-2,5-diphenyltetrazolium bromide (MTT) (Sigma-Aldrich, St Louis, MO, USA) assay as described previously [38,39,40].

### 4.5. Three-Dimensional (3D) Culture VM Tube Formation Assay

VM tube formation was assessed as described previously [23,41]. Cells (3.6 × 10^5^) were seeded on a matrigel-polymerized 24-well plate and then treated with serum with or without MiA for 16 h at 37 °C. In siRNA-transfected cells, cells (3.6 × 10^5^) were seeded after 48 h transfection and then treated with serum. In CRISPR activation plasmid-transfected cells, cells (3.6 × 10^5^) were seeded after 48 h transfection without serum. Tubular shapes were counted after imaging using an inverted light microscope Ts2_PH (Nikon, Tokyo, Japan) at 40× magnification. 

### 4.6. Western Blot Analysis

Western blot was performed in MiA-treated cells with serum and in siRNA-transfected cells with serum for 24 h, in CRISPR activation plasmid-treated cells, and in serum-treated cells with or without wortmannin (WM, Merk, Darmstadt, Germany) for 24 h. Total proteins were isolated using RIPA buffer (Thermo Scientific, Rockford, IL, USA) supplemented with phosphatase inhibitor cocktail (Thermo Scientific, Rockford, IL, USA) and protease inhibitor cocktail (Thermo Scientific, Rockford, IL, USA). The protein samples (30–35 μg) were separated by SDS-polyacrylamide gel (8–12%) electrophoresis and then transferred onto a membrane (Pall Corporation, Port Washington, NY, USA). The membrane was incubated with the indicated primary antibodies (Table 1) overnight at 4 °C, followed by incubation with specific secondary antibodies for 2 h at room temperature (RT). Protein bands were visualized using an enhanced chemiluminescence reagent (GE Healthcare, Chicago, IL, USA) and ImageJ 1.40 g software (National Institute of Health, Bethesda, MD, USA) was used to quantify each protein band.

### 4.7. Isolation of RNA and Reverse Transcriptase Polymerase Chain Reaction (RT-PCR)

Total RNA extraction was carried out in MiA-treated or Sp1 siRNA-transfected cells using a TRIzol reagent (Invitrogen, Carlsbad, CA, USA). cDNA synthesis and PCR were performed as described previously [23]. ImageJ 1.40g software was used to quantify each PCR product band.

### 4.8. Immunofluorescence Assay

Cells were seeded on an 8-well chamber slide with serum with or without MiA. In siRNA-transfected cells, cells (7 × 10^4^) were seeded on an 8-well chamber slide and transfected with siRNA for 48 h and treated with serum. Immunofluorescence assay was performed as described previously [23]. Images were captured using an ECLIPS Ts2-FL (Nikon, Tokyo, Japan) at 400× magnification.

### 4.9. Co-Immunoprecipitation (Co-IP)

The total cell lysate (300 μg) were mixed with 0.5 μg of twist antibody (Abcam plc., Cambrige, UK) for 1 h at 4 °C and then added protein A/G agarose (Santa Cruz Biotechnology, Inc., Danvers, MA, USA) for 1 h at 4 °C. The beads were collected by centrifugation and washed 3 times with lysis buffer. The immunoprecipitated protein complexes were analyzed by Western blot.

### 4.10. Statistical Analysis

All experiments were performed at least three times. Data are shown as mean ± standard deviation (SD). All data were analyzed by one-way ANOVA followed by Tukey’s studentized range test using a GraphPad Prism software (GraphPad Software Inc., San Diego, CA, USA). Means with different letters are significantly different between groups.

## Figures and Tables

**Table 1:** Antibodies used in this study.

Antibody	Company	Dilution	Product No.
Sp1	Santa Cruz	1:1000	SC-420
β-actin	Sigma-Aldrich	1:20,000	A5316
pAKT	CST	1:3000	4060
AKT	CST	1:5000	4691
MMP-2	Abcam	1:1000	ab86607
LAMC2	Abcam	1:1000	ab96327
VE-cadherin	Abgent	1:1000	AP2724a
Twist	Abcam	1:1000	ab50887
goat anti-rabbit IgG-HRP	CST	1:5000	7074P2
goat anti-mouse IgG-HRP	Bio-Rad	1:5000	STAR120P

Santa Cruz Biotechnology, Inc. (Danvers, MA, USA); CST, Cell Signaling Technology (Beverly, MA, USA); Sigma-Aldrich (St Louis, MO, USA); Abcam plc. (Cambrige, UK); Abgent (San Diego, CA, USA).

## Data Availability

Not applicable.
